# Medical–Legal Liability and Indoor Air Pollution in Non-Industrial Environments and Perspectives for Maternal and Child Health

**DOI:** 10.3390/children12101287

**Published:** 2025-09-24

**Authors:** Ginevra Malta, Angelo Montana, Antonina Argo, Stefania Zerbo, Fulvio Plescia, Emanuele Cannizzaro

**Affiliations:** 1 Department of Health Promotion, Mother and Childcare, Internal Medicine and Medical Specialties (PROMISE), University of Palermo, 90129 Palermo, Italy; 2 Department of Biomedical Sciences and Public Health, University Politecnica delle Marche, 60126 Ancona, Italy; a.montana@univpm.it

**Keywords:** indoor air pollution, medical–legal liability, indoor environmental quality (ieq), vulnerable populations, maternal and child health

## Abstract

Indoor air pollution (IAP) has emerged as a critical yet underrecognized threat to public health, particularly in non-industrial environments such as homes, schools, and healthcare facilities. As individuals spend approximately 90% of their time indoors, exposure to indoor pollutants—such as particulate matter, volatile organic compounds (VOCs), polycyclic aromatic hydrocarbons (PAHs), and microbial contaminants—can lead to significant health risks. These risks disproportionately affect vulnerable populations, including children, the elderly, and individuals with pre-existing conditions. The effects range from mild respiratory symptoms to severe outcomes like asthma, cardiovascular diseases, and cancer. This review investigates the sources, typologies, and health effects of indoor air pollutants, with a focus on their implications for maternal and child health. In particular, children’s developing systems and higher metabolic intake make them more susceptible to airborne toxins. The study also explores the legal and regulatory frameworks surrounding indoor air quality (IAQ), emphasizing how increased awareness and scientific evidence are expanding the scope of medical–legal responsibility. Legal liabilities may arise for property owners, designers, or manufacturers when poor IAQ leads to demonstrable health outcomes. Despite growing concern, there remains a significant research gap concerning the long-term health effects of chronic low-level exposure in residential settings and the efficacy of mitigation strategies. The evolution of smart building technologies and green construction practices offers promising avenues to improve IAQ while maintaining energy efficiency. However, standards and regulations often lag behind scientific findings, highlighting the need for updated, enforceable policies that prioritize human health. This work underscores the urgency of a multidisciplinary and preventive approach to IAQ, integrating public health, environmental engineering, and legal perspectives. Future research should focus on real-time IAQ monitoring, targeted interventions for high-risk populations, and the development of comprehensive legal frameworks to ensure accountability and promote healthier indoor environments.

## 1. Introduction

The significance of indoor air quality as a determinant of health has been increasingly emphasized by organizations like the World Health Organization [1]. Indoor air pollution is responsible for a substantial number of deaths globally, highlighting the urgency of addressing this environmental health risk [2]. Modern lifestyles entail individuals spending a considerable portion of their time indoors, thereby intensifying their exposure to a complex mixture of indoor air pollutants [3]. In contemporary society, individuals spend approximately 90% of their time indoors, which elevates the potential for exposure to various indoor air pollutants, frequently at levels exceeding those found outdoors [4,5]. These pollutants encompass a wide array of substances, including particulate matter, gases (such as ozone, nitrogen dioxide, carbon monoxide, and sulfur dioxide), microbial and chemical volatile organic compounds, and environmental tobacco smoke [4]. The convergence of increased time spent indoors and the presence of diverse pollutant sources underscores the critical need for effective strategies to mitigate indoor air pollution and safeguard public health. The health risks associated with indoor air pollution are often more pronounced than those linked to outdoor pollution, especially for vulnerable populations such as children, the elderly, and individuals with pre-existing respiratory or cardiovascular conditions [6]. This is further complicated by the fact that conventional monitoring approaches often provide average pollutant concentrations over extended time frames, failing to capture the nuances of temporal and spatial variations in indoor air quality [7].

Indoor air pollution represents a significant threat to public health, with concentrations of pollutants often exceeding those found in outdoor environments [8]. This is especially concerning given that a substantial portion of individuals’ time is spent indoors. The sources of indoor air pollution are diverse, ranging from building materials and furnishings to human activities such as cooking and cleaning. The factors contributing to indoor air pollution are multifaceted, including the characteristics of the building, the materials used in its construction, the ventilation systems in place, and the activities of the occupants [9]. Inadequate ventilation, common in energy-efficient buildings, can exacerbate the problem by trapping pollutants indoors [10]. The complexities of indoor air quality management are further compounded by the interactions between indoor and outdoor environments, as well as the dynamic nature of indoor spaces. Poor indoor environmental quality in homes, schools, and workplaces has become a significant concern [11]. The health consequences of indoor air pollution are far-reaching, encompassing respiratory illnesses, cardiovascular diseases, neurological disorders, and even cancer. These effects are particularly concerning for vulnerable populations such as children, the elderly, and individuals with pre-existing respiratory conditions. Exposure to indoor air pollutants can lead to both short-term and long-term health effects, including respiratory irritation, asthma exacerbation, cardiovascular problems, and even cancer.

### 1.1. Sources and Types of Indoor Air Pollutants

Indoor environments are a complex mixture of chemical, biological, and physical pollutants. Volatile organic compounds constitute a prevalent category of indoor air pollutants, arising from a multitude of sources like paints, adhesives, and cleaning products [10]. These compounds are characterized by their high volatility, mobility, and resistance to degradation, enabling their widespread distribution within indoor spaces. Formaldehyde, benzene, and toluene are well-known examples of VOCs commonly found in indoor air [12]. These substances have the capacity to trigger an array of adverse health outcomes, spanning from sensory irritation and allergic reactions to more severe conditions like respiratory diseases and cancer. Biological contaminants, including bacteria, viruses, mold, and allergens from pets and pests, can thrive in indoor environments, especially in damp or poorly ventilated areas [13]. The presence of moisture and organic materials fosters the growth and proliferation of these biological agents. Exposure to these contaminants can trigger allergic reactions, asthma attacks, and infectious diseases. The investigation of microbial contamination in indoor environments encompasses the identification of sources, the utilization of appropriate sampling strategies, and the application of suitable analysis methods. Particulate matter, another significant category of indoor air pollutants, encompasses a diverse range of solid and liquid particles suspended in the air. Sources of particulate matter include combustion processes, such as cooking and heating, as well as outdoor air infiltration.

### 1.2. Health Effects and Vulnerable Populations

Exposure to indoor air pollutants can trigger a diverse array of adverse health outcomes, impacting both respiratory and non-respiratory systems [14]. The spectrum of these effects varies from mild and transient symptoms like irritation of the eyes, nose, and throat, to more severe and persistent conditions such as respiratory infections, asthma exacerbation, cardiovascular diseases, and even cancer. The human health implications of indoor air pollution are considerable, encompassing a spectrum of respiratory ailments, cardiovascular conditions, and neurological disorders [15]. Certain subgroups of the population, including children, the elderly, pregnant women, and individuals with pre-existing respiratory or cardiovascular conditions, exhibit heightened vulnerability to the detrimental effects of indoor air pollution. Children, owing to their developing respiratory systems and higher ventilation rates, are disproportionately affected by indoor air pollution. The intricate interplay between outdoor air pollution and indoor environments further complicates the overall exposure scenario. Individuals with pre-existing respiratory ailments, such as asthma and chronic obstructive pulmonary disease, experience heightened susceptibility to the adverse effects of indoor air pollutants, which can trigger symptom exacerbation and diminished lung function [13]. Long-term exposure to indoor air pollution has been linked to the development of chronic respiratory diseases, cardiovascular problems, and even cancer [16].

### 1.3. Legal and Regulatory Frameworks

The medical–legal ramifications of indoor air pollution are evolving, as scientific evidence increasingly links indoor air quality to specific health outcomes. The evolving comprehension of the intricate correlation between indoor air quality and human health is progressively underscoring the legal and regulatory facets of this pervasive environmental challenge. In cases where exposure to indoor air pollutants can be directly linked to adverse health effects, legal claims may arise based on negligence, breach of warranty, or product liability. The establishment of causation between indoor air pollution exposure and specific health outcomes is a complex endeavor, often requiring extensive scientific and medical evidence. Occupants of residential or commercial properties may pursue legal recourse against landlords, property managers, or building owners for failing to maintain safe and healthy indoor environments. Building codes and standards play a crucial role in regulating indoor air quality in new construction and renovations. These codes and standards often specify ventilation requirements, material emissions limits, and other measures to minimize indoor air pollution.

Continued vigilance remains essential in mitigating the effects of air pollution on human health and pulmonary well-being [17]. It is vital to consider air quality in multi-purpose halls, especially the interactions between thermal comfort, energy consumption and ventilation [18]. With individuals spending approximately 90% of their time indoors, maintaining healthy indoor environments is crucial [19]. Mitigating negative factors associated with air quality issues involves careful consideration of various factors. The chemical components of indoor environments, particularly those with toxic properties, are of particular concern for building occupants’ health [9]. Smart and sustainable approaches to green building construction should incorporate indoor air quality as a critical component of building design [20].

An integrated approach is essential for managing residential indoor air quality, emphasizing the interconnectedness of various factors [21]. IAQ surveys are essential for characterizing human exposure and addressing concerns, encompassing contextual information to elucidate sources, pathways, and exposure magnitude [5]. Comprehensive investigation of indoor air quality constitutes a pivotal stride towards evaluating the well-being of indoor air, especially before a newly adorned space is inaugurated for utilization [22]. The effectiveness of implemented control measures should be evaluated through post-intervention IAQ assessment.

Ventilation systems, along with air extractors and purifiers, have been developed to deliver good air quality and ensure occupant health and comfort [21]. IAQ is greatly affected by ventilation within an indoor environment, but also by other factors such as household chemicals, furnishings, air contaminants emitted from outside, and occupant activities [23]. Furthermore, numerous studies have indicated that even levels of CO_2_, that pose no health risks, can still affect human intellectual productivity [24]. An unhealthy environment can lead to sick building syndrome and building-related illnesses [2,25]. The emphasis should be on developing optimally informative methods for quantifying these effects, to ensure indoor air is pleasant and has no adverse effects on health or human performance [26].

Recent developments in European legislation further emphasize IAQ protection. The 2020 European Green Deal and the 2021 WHO Air Quality Guidelines underline the importance of stricter IAQ standards in public and private buildings. In Italy, the 2022 National Recovery and Resilience Plan (PNRR) introduced mandatory IAQ monitoring in newly renovated schools and healthcare facilities. At the international level, cases before the European Court of Human Rights (2022–2023) have expanded the interpretation of the “right to health” to include protection against harmful indoor exposures. These regulatory and judicial updates demonstrate how liability is increasingly framed in terms of the duty to protect vulnerable populations, particularly children, pregnant women, and the elderly [27].

### 1.4. Case Studies of Medical–Legal Liability Related to Indoor Air Quality

Recent case law highlights how poor indoor air quality has become a ground for legal claims in both civil and administrative courts. For instance, in the United States, litigation concerning mold contamination in residential buildings has led to compensation for respiratory conditions and allergic reactions among tenants. In Europe, Italian courts have addressed cases involving inadequate school ventilation, recognizing municipal liability for exacerbations of asthma in children. Similarly, in France, parents have successfully claimed damages related to exposure to asbestos and radon in public schools. These examples illustrate the growing judicial sensitivity to IAQ as a determinant of health and the potential expansion of liability to landlords, school administrators, and public authorities [11,28].

## 2. Literature Review

Research has demonstrated that simulated indoor environmental quality conditions in green buildings have an impact on cognitive function [29]. These findings suggest that optimizing indoor environmental quality in green buildings may enhance cognitive performance. A review of published research on the effects of green buildings indicates that they provide healthier environments for occupants through the creation of healthy indoor environments and the minimization of negative environmental impacts [30]. Studies have explored the effects of passive design strategies on building ventilation performance and thermal comfort, finding that increasing window-to-wall ratio percentages can enhance ventilation efficiency and indoor thermal comfort [31]. However, in urban areas, indoor environmental quality has been declining due to factors such as inadequate building ventilation, poor ambient air quality, and the usage of solid fuels for cooking [31].

Effective heating, ventilation, and air conditioning systems are necessary to maintain acceptable indoor comfort and air quality [32]. However, these systems can account for a significant portion of building energy consumption. Therefore, the design and operation of HVAC systems should balance energy efficiency with the provision of adequate ventilation and air filtration. Traditional approaches to indoor environmental quality often lead to energy overconsumption, as systems operate on fixed schedules or broad setpoints rather than responding to real-time occupant needs [33]. Smart building technologies offer the potential to optimize indoor environmental quality while minimizing energy consumption. Advanced sensing and control systems can monitor indoor conditions and adjust HVAC operations in response to occupancy patterns, weather conditions, and air quality levels.

These technologies enable demand-controlled ventilation, where ventilation rates are adjusted based on real-time occupancy levels and air quality measurements, reducing energy waste. Furthermore, green and sustainable building programs are driving the trend towards sustainable buildings, and the manner in which they consider IAQ is critical to achieving energy-efficient buildings with good indoor environments [34]. Finding strategies for achieving a satisfactory balance between good indoor air quality and rational energy use is a priority for researchers and designers [35]. The urgency to identify and mitigate indoor air pollution sources arises from the extended periods individuals spend indoors. Ventilation is a key strategy for improving IAQ by diluting indoor air pollutants and providing fresh air [2]. However, ventilation rates must be carefully controlled to avoid excessive energy consumption.

Air-side design plays a crucial role in achieving optimal air quality and thermal comfort in healthcare facilities [36]. Air-conditioning systems should be correctly designed, maintained, and operated to minimize energy consumption and provide acceptable thermal comfort and indoor air quality. Natural ventilation methods are frequently used to maintain IAQ in places with limited resources by opening windows and doors [37]. When designing educational buildings, architects often prioritize functional requirements, but sustainable educational environments require consideration of natural ventilation design measures [38]. Studies show that sufficient ventilation rates and acceptable indoor air quality contribute to enhancing students’ health and learning performance [39]. The operation of mechanical ventilation and air conditioning systems is critical because they directly affect both the indoor environment and energy consumption of buildings.

Indoor air quality monitoring is essential for maintaining healthy buildings, offering a continuous data stream for centralized regulation of building automation procedures [40].

Modern HVAC systems play an important role in ensuring indoor air quality for safety and comfort, but they also consume a significant portion of total energy usage in buildings [41]. In the context of residential buildings, IAQ is influenced by factors such as outdoor air quality, ventilation, combustion appliances, building materials, and occupant activities. The American Society of Heating, Refrigerating and Air-Conditioning Engineers defines acceptable indoor air quality as air in which there are no known pollutants at harmful concentrations, and where a majority of occupants do not express dissatisfaction with thermal comfort, noise, vibrations, or lighting [42]. Architects and engineers are increasingly being held liable for IAQ issues, resulting in financial losses and reputational damage [19]. The significance of indoor environmental quality encompasses improvements to occupants’ comfort and productivity by regulating the indoor environment. This regulation often involves controlling variables like indoor air quality, thermal comfort, lighting, and acoustics [31,43,44,45]. Among the elements of IEQ, thermal factors are closely related to occupants’ thermal comfort, health, productivity, and thermal sensation [46]. Thermal comfort is a subjective feeling that is influenced by personal and environmental factors, affecting satisfaction with the thermal environment [47]. Ventilation plays a key role in preventing air contamination and overheating, thus ensuring that spaces are conducive for human activities [48].

Indoor environmental quality can significantly affect student learning satisfaction, emphasizing the importance of identifying the extent of classroom physical IEQ in educational settings [49]. The indoor environment is affected by flooring materials, windows, the outdoor environment, ventilation systems, and the occupants and their activities [50]. People spend most of their time indoors, making them more vulnerable to indoor environmental pollution from various sources, including radon, formaldehyde, volatile organic compounds, and airborne particles [51,52]. Attention from the international scientific community, political institutions, and environmental governances regarding Indoor Air Quality has increased in the last few decades [53]. It is widely recognized that poor indoor air quality has significant adverse effects on human health, with a wide range of symptoms like fatigue and respiratory problems [54]. Effective indoor environmental quality can be improved by adjusting parameters to improve satisfaction and well-being [55]. The symptoms are often linked to inadequate indoor environment, like itchy, headache, fatigue, and respiratory issues such as cough and congestion [54].

Standards typically address environmental factors such as thermal comfort, indoor air quality, and aural and visual environments separately, but these factors have combined effects on occupants’ acceptability and work performance [56]. The quality of the office environment significantly impacts work efficiency and worker health, both of which contribute to a company’s overall performance [57]. IAQ guidelines are designed to balance energy conservation, thermal comfort, and good IAQ [58].

### 2.1. Search Strategy

#### 2.1.1. Datasets

Scopus, Web of Science and PubMed were explored using keywords.

Keywords: indoor air quality AND law, indoor air quality AND liability, indoor air quality AND legal, indoor air quality AND compensation, indoor air quality AND non-industrial.

#### 2.1.2. Inclusion and Exclusion Criteria

The search was conducted for articles published in English. Articles were included if they were centered around indoor air pollution in non-industrial environments and the related medical–legal liabilities. Studies were excluded if they were related to industrial settings or were not written in English.

#### 2.1.3. Study Selection

The final selection included research articles that addressed indoor air quality issues in residential, commercial, or public buildings, and explored the connection between these issues and potential legal liabilities.

#### 2.1.4. Data Extraction

Key data extracted included the types of indoor pollutants investigated, the health effects associated with these pollutants, and the legal frameworks discussed in relation to indoor air quality.

## 3. Results

Recent trends in college campus construction emphasize sustainable designs [59]. These buildings are designed and operated to provide high-quality environments, including indoor air quality [60].

Maintaining good indoor air quality is crucial, as people spend a significant amount of time indoors, making them susceptible to health issues related to indoor pollutants [61]. A comprehensive review of factors influencing air pollution in residential buildings highlights that people spend approximately 90% of their time in indoor environments, where contaminants are produced through activities like heating, cooling, and cooking [62]. Individuals spend a large proportion of their time in offices, educational institutes, and other commercial buildings [62]. These indoor environments can harbor a variety of pollutants, including particulate matter, volatile organic compounds, and biological contaminants [13].

The effect of indoor air quality on people is significant because individuals spend approximately 90% of their time indoors, such as in homes, offices, and industrial buildings [63]. People spend more than 80% of their time in confined environments; thus, it is necessary to evaluate indoor contaminant concentrations and distributions for assessing total human exposure to them [64]. Poor indoor air quality can lead to various health issues, ranging from mild discomfort to severe diseases [65]. Improvements in indoor air quality through higher ventilation rates have been associated with reduced prevalence of sick building syndrome symptoms, respiratory infections, asthma symptoms and absenteeism [66]. Recent research has focused on the complex interactions between occupant health and indoor spaces, shifting from studying discrete pollutant sources to comprehensive assessments [67].

A critical appraisal of the reviewed studies reveals substantial heterogeneity in study designs, which influences the strength and generalizability of their findings. Many studies are observational or cross-sectional, often with small sample sizes, limited follow-up, and potential selection bias, which restrict their capacity to infer causality. Laboratory simulations and modeling studies, while offering controlled conditions and mechanistic insights, often lack real-world exposure variability and ecological validity. Conversely, large-scale epidemiological investigations, such as recent works from the EDIAQI project, provide robust population-level data but sometimes rely on self-reported exposure metrics and are vulnerable to residual confounding. By explicitly distinguishing between study designs and highlighting their methodological limitations, the present review aims to contextualize the evidence base rather than present findings as equally robust. Future research should prioritize longitudinal and multi-site studies integrating real-time monitoring and standardized IAQ metrics to improve the reliability and comparability of results.

This shift acknowledges that the confluence of multiple environmental factors can have synergistic impacts on health [66]. There is substantial knowledge on individual factors and their effects, though understanding how factors interact and what role occupants play in these interactions is lacking [68]. The household environment should sustain the child, stimulate activity, support the child’s self-sustaining capacities, and control the quantity and pattern of experiences for the child [69]. Children are far more sensitive than adults to toxic chemicals in the environment [70]. Children have a higher rate of exposure because they breathe faster, drink more fluids and eat more food per kilogram of body weight than adults. Children’s developing organ systems are more vulnerable, and they have less developed detoxification systems [71].

Examining the sources of indoor air pollution, combustion processes, building materials, and human activities contribute to poor indoor air quality [72]. Inadequate ventilation exacerbates the problem, leading to the accumulation of pollutants and potential health risks [73]. Indoor air quality is influenced by physical factors like dust particles and gaseous pollutants, as well as biological factors like molds and bacteria, which depend on temperature and humidity [74]. It is a significant risk factor for the health of the general population because humans spend a considerable amount of their time breathing air inside enclosed spaces [75]. Exposure to fine particulate matter is a major contributor to the global human disease burden [76]. The sources of indoor air pollution include emissions from building materials, combustion appliances, and human activities like cooking and cleaning [77]. Volatile organic compounds and particulate matter are common air pollutants, with sources ranging from smoking and cooking to household products and building materials [10]. The most rapid increase in particle concentration was during thermal source episodes such as candle, cigarette, incense stick burning, and cooking-related sources, while the slowest decay of concentrations was associated with sources, emitting ultrafine particle precursors, such as furniture polisher spraying, floor wet mopping with detergent etc. [3,78,79]. The physical and chemical phenomena that control pollutant transport and transformations outdoors differ from those that are important indoors, due to factors such as shorter air residence times indoors, altered abundances of oxidative species, and higher surface-to-volume ratios in indoor environments compared to urban and regional atmospheres [80]. Outdoor air pollutants can infiltrate indoor environments, further affecting indoor air quality, particularly in urban and industrialized areas [81]. Indoor air quality is also affected by outdoor pollutant concentrations [81]. Air pollutants rarely exist singly, but the combined pollutants may have synergistic, additive or antagonistic effects [82].

The legal framework governing indoor air quality (IAQ) varies significantly across jurisdictions, with marked implications for enforcement and liability [83,84]. In the European Union, IAQ protection has been progressively incorporated into the European Environment Agency directives and the World Health Organization 2021 IAQ Guidelines, which set health-based exposure limits and recommend periodic monitoring in public buildings. In Italy, the 2022 National Recovery and Resilience Plan (PNRR) introduced mandatory IAQ monitoring in schools and healthcare facilities, explicitly linking non-compliance to administrative liability for local authorities. By contrast, the United States Environmental Protection Agency (EPA) issues IAQ standards as non-binding recommendations, and enforcement primarily occurs through litigation, as seen in cases of mold contamination and radon exposure where courts have imposed compensatory damages on landlords and school boards. French and Italian courts have recently recognized municipal liability for inadequate ventilation systems in schools leading to asthma exacerbations in children, while U.S. courts have applied product liability doctrines against manufacturers of building materials releasing hazardous compounds indoors. This comparative analysis highlights how differing legal mechanisms (regulatory enforcement vs. tort litigation) shape the thresholds for liability, and underscores the need for harmonized IAQ regulations to protect vulnerable populations and reduce litigation-related uncertainty [85,86,87,88].

## 4. Discussion

**Figure 1:**
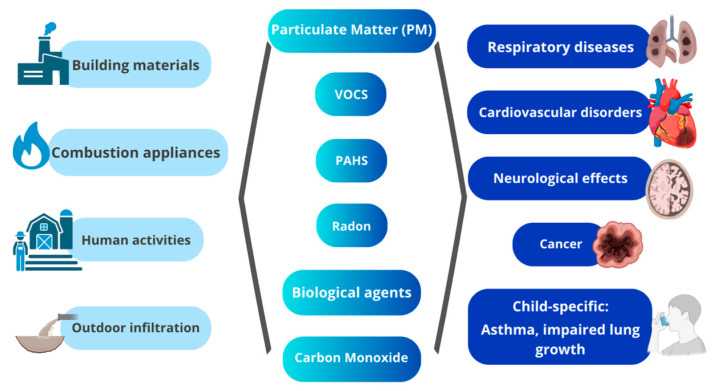
Interaction between Pollutants and the Human Body.

People’s health and well-being are greatly impacted by indoor air quality; therefore, maintaining acceptable air quality indoors is essential to protect public health and safety. As more and more people move into cities, research on air quality control has switched its focus from outdoor to indoor settings. A closer look at the health consequences of non-industrial indoor air pollution reveals that those who are already vulnerable are more likely to experience negative effects from long-term exposure to low levels of indoor air pollutants [4]. It is becoming increasingly obvious that polycyclic aromatic hydrocarbons may have an impact on processes related to non-malignant airway illnesses [89]. Research is also being conducted on the effects of exposure to numerous indoor air pollutants and their possible interactions (Figure 1, Table 1) [4].

Because they breathe more quickly, consume more fluids, and consume more food per kilogram of body weight than adults do, children are more vulnerable to toxic substances in the environment. Children are more vulnerable because their organ systems are still developing and their detoxification systems are less advanced. Legal frameworks must take into account the particular requirements of vulnerable populations like children, the elderly, and those with pre-existing respiratory disorders.

Exposure to airborne polycyclic aromatic hydrocarbons leads to adverse health outcomes [90]. The incomplete combustion of fossil fuels, wood, and tobacco results in the ubiquitous environmental pollutants known as polycyclic aromatic hydrocarbons [91]. Exposure to polycyclic aromatic hydrocarbons has been linked to a number of health issues, including respiratory illnesses, cardiovascular problems, and cancer [92].

The complex mixture of contaminants found in indoor air must be characterized using cutting-edge analytical techniques in order to fully understand the risks to one’s health. The sources and dynamics of particle-bound PAHs must be identified using receptor models, which will aid in the creation of effective mitigation strategies. The need to create effective indoor air quality management strategies is highlighted by the fact that air pollution inside is a complicated and diverse problem.

## 5. Limitation

A major drawback is that there are not many studies that look into the health consequences and legal ramifications of indoor air pollution in non-industrial environments. Future research should concentrate on examining the long-term health consequences of exposure to low levels of indoor air pollutants, particularly in vulnerable populations like children and the elderly [6].

Further research is needed to evaluate the efficacy of various intervention strategies for reducing indoor air pollution and promoting healthier indoor environments [93].

## 6. Conclusions

Indoor air pollution in non-industrial settings presents a multifaceted challenge with significant medical–legal implications, especially given that people spend over 90% of their time indoors, where pollution levels often exceed those outdoors [7]. Indoor air pollution is a major public health concern, especially for vulnerable populations [5]. It is responsible for a substantial number of deaths worldwide annually [2]. Increased awareness of indoor air pollution emphasizes the significance of the interactions among pollutants, health consequences, and legal frameworks [10]. Further research is needed to fully understand the complex interactions between indoor air pollutants and human health, as well as to develop effective strategies for mitigating indoor air pollution and promoting healthier indoor environments [10,94].

## Figures and Tables

**Table 1:** Interaction between Pollutants and Human Body.

Pollutant	Main Sources	Health Effects (Especially in Children)
Particulate Matter (PM2.5, PM10)	Cooking, heating, smoking, candles, outdoor infiltration	Asthma, bronchitis, impaired lung development, cardiovascular risk
Volatile Organic Compounds (VOCs: benzene, toluene, formaldehyde)	Paints, adhesives, cleaning agents, furniture	Irritation of eyes and respiratory tract, neurotoxicity, cancer
Polycyclic Aromatic Hydrocarbons (PAHs)	Combustion of fossil fuels, tobacco smoke, fireplaces, incense	Asthma, airway inflammation, lung cancer, cardiovascular diseases
Radon	Soil infiltration, building materials	Lung cancer
Biological contaminants (mold, bacteria, viruses, allergens)	Damp environments, poor ventilation, pets, pests	Allergies, asthma exacerbations, respiratory infections
Carbon Monoxide (CO)	Stoves, heaters, fireplaces, tobacco smoke	Headaches, neurological effects, death at high levels

## Data Availability

The data are not available due to [ethical/legal/commercial] restrictions, and in addition the participants in this study signed a written consent where they were guaranteed to anonymize their data to be shared publicly, so due to the sensitive nature of the research the supporting data are not available.
